# *In vitro* Effects of Bacterial Exposure on Secretion of Zonulin Family Peptides and Their Detection in Human Tissue Samples

**DOI:** 10.3389/fmicb.2022.848128

**Published:** 2022-04-14

**Authors:** Ching Jian, Sonja Kanerva, Sami Qadri, Hannele Yki-Järvinen, Anne Salonen

**Affiliations:** ^1^Human Microbiome Research Program, Faculty of Medicine, University of Helsinki, Helsinki, Finland; ^2^Minerva Foundation Institute for Medical Research, Helsinki, Finland; ^3^Department of Medicine, University of Helsinki and Helsinki University Hospital, Helsinki, Finland

**Keywords:** zonulin, ELISA, bacterial exposure, gut microbiota, *Lactobacillus rhamnosus* GG, liver

## Abstract

Commercially available ELISAs for zonulin (pre-haptoglobin 2), a protein with tight junction regulatory activity in the epithelia, were recently shown to recognize other proteins that are structurally and functionally related to zonulin, termed zonulin family peptides (ZFPs). With little or no information about the identity and property of ZFPs, various commercial zonulin ELISA kits are widely utilized in research as a marker of intestinal permeability. Bacterial exposure is a known trigger for the secretion of zonulin, but it remains unclear whether distinct bacteria differ in their capability to stimulate zonulin secretion. We hypothesized that ZFPs are similar to zonulin regarding response to bacterial exposure and aimed to compare the effects of non-pathogenic, Gram-negative bacteria (*Escherichia coli* RY13 and *E. coli* K12 DH5α) and probiotic, Gram-positive bacteria (*Lactobacillus rhamnosus* GG and *Bifidobacterium bifidum*) on ZFP secretion in an *in vitro* model. Additionally, utilizing samples from human clinical trials, we correlated circulating levels of ZFPs to the gut bacteria and determined the presence of ZFPs in various human tissues. Unexpectedly, we found that the ZFPs quantified by the widely used IDK® Zonulin ELISA kits are specifically triggered by the exposure to live *Lactobacillus rhamnosus* GG in HT-29 cells, associated with absolute abundances of intestinal *Lactobacillus* and *Bifidobacterium* in adults, and are copious in the small intestine but undetectable in the liver or adipose tissue. These characteristics appear to be different from zonulin and highlight the need for further characterization of ZFPs recognized by commercially available and widely used “zonulin” ELISAs.

## Introduction

Intestinal barrier dysfunction, characterized by increased intestinal permeability, is a key pathological mechanism potentially underlying several diseases that feature the inflammatory host–microbiome crosstalk (Vancamelbeke and Vermeire, 2017).

Zonulin, identified as pre-haptoglobin 2 (Tripathi et al., 2009), is a mammalian analog of the zonula occludens toxin (Zot) of *Vibrio cholera*. Similar to the bacterial toxin, zonulin released from the intestinal lamina propria leads to reversible disassembly of the intercellular tight junctions *via* receptor-activated intracellular cytoskeleton reorganization (Fasano, 2020), increasing intestinal permeability. Circulating levels of zonulin have been frequently measured as a proxy of intestinal permeability and linked to various diseases in human studies (Fasano, 2020). Almost all the studies measuring zonulin have utilized commercially available ELISAs, as opposed to the in-house assays exclusively used by the researchers who discovered zonulin (Wang et al., 2000). The identity of zonulin quantified by different assays and its correlation to functional intestinal permeability tests, such as the lactulose-mannitol test, have been extensively debated recently (Scheffler et al., 2018; Ajamian et al., 2019; Tatucu-Babet et al., 2020; Fasano, 2021; Massier et al., 2021; Power et al., 2021; Seethaler et al., 2021; Sollid and Koning, 2021). To reconcile the discrepancies, it was proposed that the commercially available zonulin ELISAs recognize other proteins that are structurally and functionally related to the zonulin protein originally described by Dr. Alessio Fasano (Wang et al., 2000), termed zonulin family peptides (ZFPs; Fasano, 2021). Therefore, measurements by commercially available zonulin ELISAs in this and earlier studies are referred as ZFPs herein. Much remains unknown about zonulin as well as ZFPs, especially regarding their expression and secretion. There is ample evidence on the increase of serum ZFPs in obesity, non-alcoholic fatty liver disease coronary artery disease, type 2 diabetes, and polycystic ovary syndrome; in fact, it has been suggested that circulating ZFPs are rather a marker of metabolic disease than intestinal permeability (Ohlsson et al., 2017).

The main triggers of zonulin secretion from intestinal cells that have been described so far are gliadin, the key protein in inflammatory response to gluten in celiac disease, and bacteria (El Asmar et al., 2002; Clemente et al., 2003). The specific mechanism behind bacteria-induced zonulin or ZFP secretion is unclear. Early work suggests that exposure of Gram-negative bacteria irrespective of their virulence lead to increased secretion of zonulin, concomitant to a decrease in transepithelial electrical resistance in *ex vivo* mammalian small intestines and intestinal cell monolayers (El Asmar et al., 2002). On the other hand, several Gram-positive probiotic strains have been shown to counteract gliadin-induced ZFP secretion and epithelial barrier disruption *in vitro* (Orlando et al., 2014; Xu et al., 2016), although the effects of those Gram-positive bacteria on ZFP secretion in the absence of gliadin are unclear. In addition, cross-sectional human studies have reported significant associations between serum ZFPs and the abundance of total bacteria (Zak-Gołąb et al., 2013) or specific bacterial taxa (Mokkala et al., 2016; Mörkl et al., 2018) in the gut microbiota.

In the present study, we hypothesized, based on the earlier studies, that different Gram-negative and Gram-positive bacteria possess variable capability to stimulate ZFP secretion. We investigated the effects of non-pathogenic, Gram-negative bacteria (*Escherichia coli* RY13 and *E. coli* K12 DH5α) and probiotic, Gram-positive bacteria (*Lactobacillus rhamnosus* GG and *Bifidobacterium bifidum*) on the secretion of ZFPs measured by widely used IDK® Zonulin ELISA kits (Supplementary Table 1) in intestinal HT-29 cell cultures. The *in vivo* associations between circulating ZFPs and total bacteria, *Bifidobacterium*, *Lactobacillus*, and *E. coli*, in the gut microbiota were examined in a cohort of adults with overweight or obesity. Additionally, we attempted to determine the presence of ZFPs in the human liver, jejunal, and subcutaneous adipose tissues.

## Materials and Methods

### Bacterial Strains and Culture Conditions

*Lactobacillus rhamnosus* GG (LGG; ATCC 53103) was obtained from the Valio culture collection (Valio Ltd., Helsinki, Finland). *Bifidobacterium bifidum* (DSMZ 20456), *E. coli* RY13, and *E. coli* K12 DH5α were available internally. Prior to experiments, LGG (ATCC 53103) and *B. bifidum* (DSMZ 20456) were cultured at 37°C on Man-Rogosa-Sharep agar (Lactobacilli MRS Broth, Difco, France) supplemented with L-cysteine (Sigma Aldrich, Germany) for 24–48 h under anaerobic conditions. Liquid cultures of LGG and *B. bifidum* were then prepared from single colonies using MRS broth (Lactobacilli MRS Broth, Difco, France) supplemented with L-cysteine. The *E. coli* strains were cultured at 37°C on Luria agar (Sigma Aldrich, United States) overnight and colonies were picked for liquid culture in Luria-broth (Sigma Aldrich, USA). Heat treatment of the bacterial strains was performed by heating at 65°C for 0.5 h. The success of heat-killing was confirmed by plating the heat-killed bacteria on MRS or Luria agar overnight at 37°C.

### Cell Culture

The human colonic epithelial HT-29 cells (DSMZ, ACC 299) were cultured in McCoy’s 5A medium containing 10% FBS and 100 U/ml antibiotics (Pen-Strep). Cells were cultured at 37°C in an atmosphere of 95% air and 5% CO_2_ and passaged 10^5^ cm^−2^ when reaching 70%–90% confluence.

### Treatment of HT-29 Monolayers With Bacteria

A 5 × 10^5^ HT-29 cells were plated and incubated as described above. Upon reaching a confluency of 70%–80%, the cells were washed twice with PBS and the growth medium was replaced with serum-free McCoy’s 5A medium without any supplements. The cells were incubated in the serum-free medium for 24 h prior to use. The overnight bacterial liquid cultures (LGG, *B. bifidum*, *E. coli* RY13, and *E. coli* DH5α) were diluted 1:100 in the corresponding growth media and cultured until reaching an OD_600_ of 0.66–0.67. Serial dilutions of the cultured bacteria were prepared and plated on agar for viable counting. An OD_600_ of 0.66–0.67 corresponded to *ca.* 5 × 10^8^ colony forming units per milliliter (CFU/ml). An aliquot of 4.8 ml/strain was collected and the bacteria recovered by centrifugation at 3,000 rpm for 5 min. The pelleted bacteria were washed once with PBS and re-suspended in 24 ml of serum-free cell culture medium without antibiotics to obtain *ca.* 10^8^ bacteria/ml.

The bacterial suspension was added to the apical side of the cell monolayers and incubated at 37°C with 5% CO_2_ for 16 h. Previous studies in HT-29 and Caco-2 cells showed that the concentrations of ZFPs typically peaked from 5 to 20 h after bacterial exposure (Li et al., 2016; Xu et al., 2016). Therefore, the exposure duration of 16 h was chosen to allow adequate ZFP secretion from the cells while avoiding potential cytotoxic effects following prolonged bacterial exposure. Serum-free medium without bacteria and antibiotics was used as a negative control. The cell culture medium was collected at 16 h and stored at −20°C until analysis.

### Human Cohorts and Collection of Fecal, Serum, and Tissue Samples

Serum and fecal samples were derived from 38 adults with overweight or obesity (age 48 ± 2 years, BMI 31 ± 1 kg/m^2^) enrolled in a clinical trial comparing effects of different diets on intrahepatic triglycerides (IHTGs; Clinical trial reg. no. NCT02133144, clinicaltrials.gov). Cohort characteristics are summarized in Supplementary Table 2 and described in detail elsewhere (Luukkonen et al., 2018). To increase statistical power, all the samples (76 serum and fecal samples collected at two time points of the trial) with available data on the concentrations of serum ZFPs and the corresponding gut microbiota were included in the present study. The jejunal section from the resected intestine (*N* = 1), as well as liver (*N* = 4) and subcutaneous adipose tissue (*N* = 1) biopsies were taken from obese subjects during gastric bypass surgery (Qadri et al., 2020). Measurement of IHTGs was performed by proton MRS (^1^H-MRS; Luukkonen et al., 2018). The study protocols were approved by the Medical Ethical Committees of the Hospital District of Helsinki and Uusimaa and HUCH.

### Gut Microbiota Analysis From Fecal Samples

Previously, generated data on the gut microbiota from the fecal samples (Jian et al., 2021) were used for correlation analysis in the present study. Briefly, bacterial DNA from the fecal samples of the 38 participants collected at two time points (total *N* = 76) was extracted. Illumina MiSeq paired-end sequencing of the hypervariable V3–V4 regions of the 16S rRNA gene (primers 341F/785R) was performed (Luukkonen et al., 2018; Jian et al., 2021); the sequence files are available in the European nucleotide Archive under accession number PRJEB35994. For this study, enumeration of total bacteria was carried out by qPCR with the universal 331F/797R primers, and absolute abundances of genus-level data were estimated and 16S rRNA gene copy-number corrected as previously described (Jian et al., 2020).

### Measurement of ZFPs in Cell Culture Media and Serum Samples

Zonulin family peptides in cell culture media and serum samples were quantified by IDK® Zonulin (Serum) ELISA (K5601, Immundiagnostik AG, Bensheim, Germany) according to manufacturer’s instructions. The IDK® Zonulin ELISA kits were used in more than 40% of the recently published studies and therefore were chosen among *ca.* 20 different brands currently offering zonulin ELISA kits (Supplementary Table 1). The specificity and comparability between different brands are outside the scope of this study. The cell culture media were clarified by centrifugation (5,000 rpm, 2 min; Hettich zentrifugen MIKRO 185, Germany), and supernatants were concentrated (4x) with a SpeedVac Plus Vacuum concentrator (Savant SC110A) for the analysis. The intra-assay coefficient of variation was <10%. The limit of quantitation for the ELISA is 0.183 ng/ml.

### Measurement of ZFPs in Human Tissues

Jejunal, liver, and subcutaneous adipose tissue biopsies (10–50 mg) were added to 300 μl of lysis buffer designed to retain protein tertiary structure containing PBS (pH 7.4), 0.5% Tween-20, and protease inhibitor (Thermo Fisher). The lysis buffer had no effect on measurement of ZFPs when tested using control serum samples (Supplementary Figure 1). The mixture was placed in a bead tube containing 2.5 mm ceramic beads and 0.1 mm zirconia beads, and homogenized by FastPrep®-24 five times at 4°C for 1 min (5 m/s). The tissue lysate was centrifuged at 15,000 *g* at 4°C for 20 min to precipitate cell debris. The supernatant was transferred to a new tube, and the centrifugation step was repeated. Cleared tissue lysates were diluted 10 times and used to determine ZFP content by IDK® Zonulin (Stool) ELISA (K5600, Immundiagnostik AG, Bensheim, Germany). The stool kit was used for the ZFP measurement in tissue lysates as it was designed to measure ZFPs in complex matrices and showed highly comparable readouts to the serum kit when control serum samples were tested (Supplementary Figure 1). The intra-assay coefficient of variation was <10%. The limit of quantitation for the ELISA is 0.188 ng/ml. Total protein content in the tissue lysates was determined by the bicinchoninic acid (BCA) assay (Pierce Biotechnology, Rockford) and used to normalize the ZFP signal.

### Statistical Analysis

The Wilcoxon rank-sum test was used to determine between-group differences, i.e., the differences in ZFP concentrations between different bacterial treatments *in vitro*. Spearman’s rank correlation coefficients (expressed as *R*) were used to assess associations. Values of *p* < 0.05 were considered significant. Statistical analyses were performed with the statistical program R version 3.5.0 and RStudio version 0.99.903.

## Results

**Figure 1:**
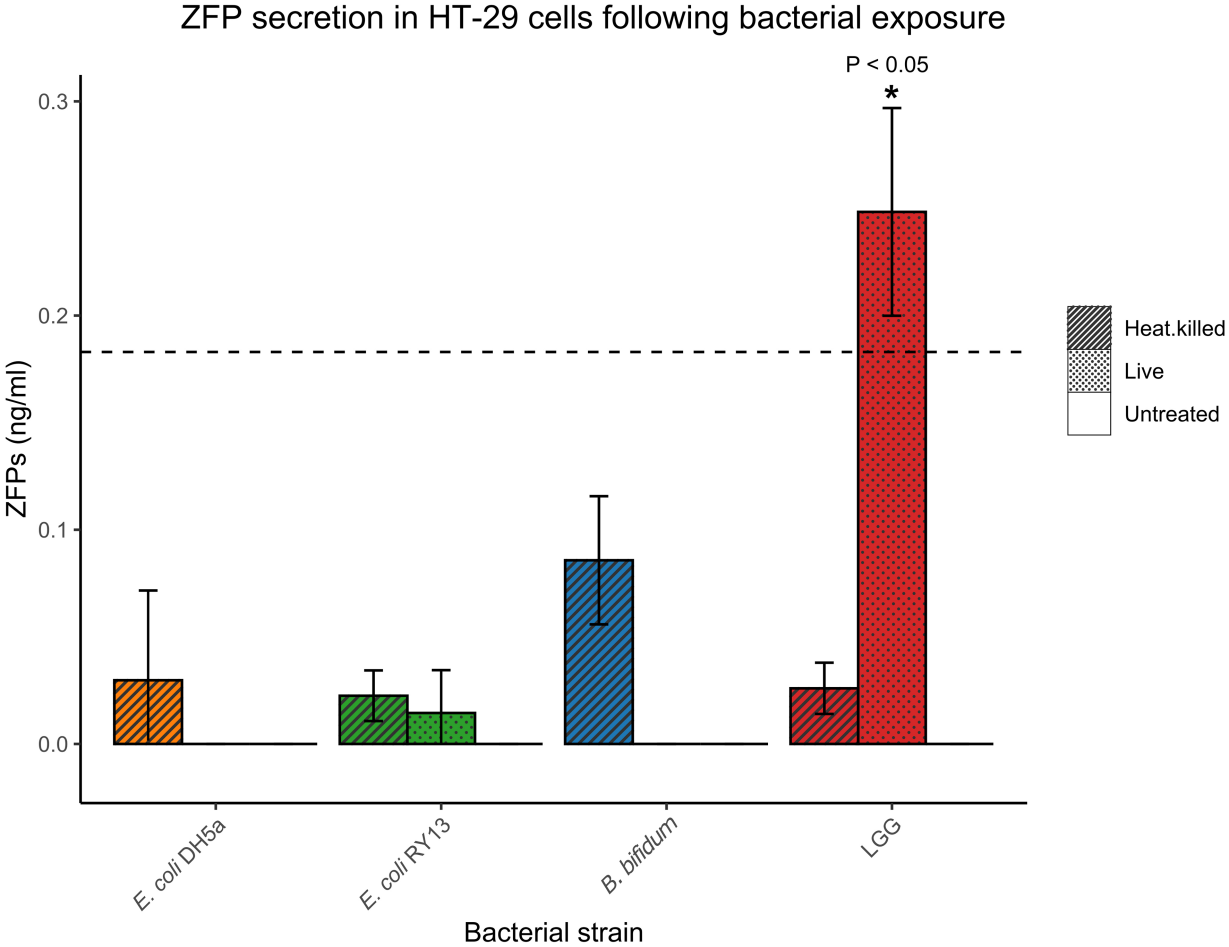
Concentrations of zonulin family peptides (ZFPs) in HT-29 cell media after exposure to various bacterial strains after 16 h. Error bars represent SDs of three technical replicates in the experiments. The dash line indicates limit of quantification (LoD) of the ELISA kit. Statistical significance compared to untreated controls is designated with asterisks (^*^p < 0.05).

Basal concentrations of ZFPs secreted by HT-29 cells were generally low or undetectable. After the 16-h incubation period, only the cells exposed to live LGG were above detection limit and had significantly elevated levels of ZFPs compared to the untreated cells [0.25 ± 0.05 ng/ml (mean ± SD), *p* < 0.05; Figure 1].

**Figure 2:**
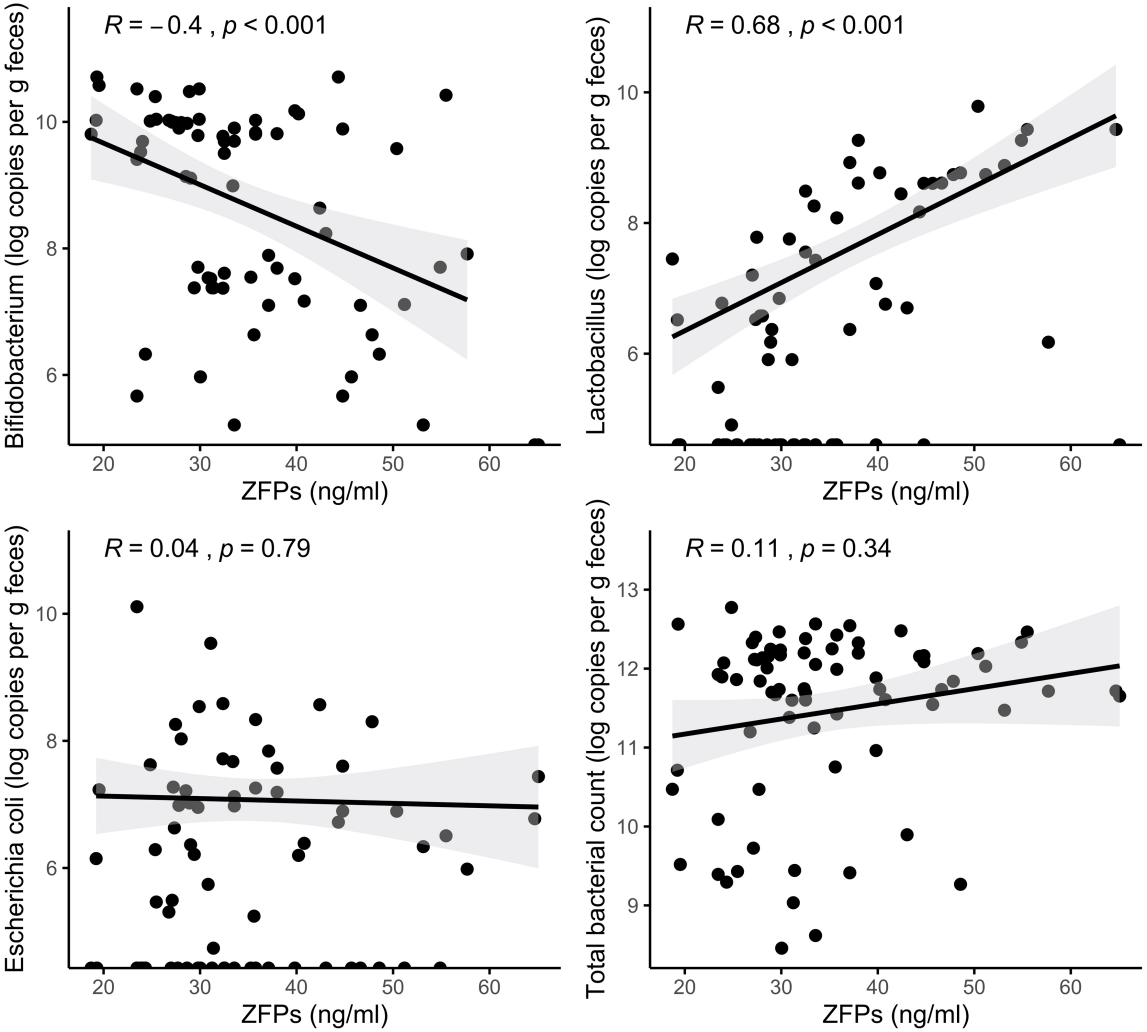
Associations between serum ZFPs and gut bacteria quantified from fecal samples. The lines represent the fitted regression lines (Spearman’s rank correlation coefficients and *p* values displayed at the upper left corner) and the corresponding shaded area represents the 95% CIs.

To further study the potential relationship between circulating ZFPs and the gut microbiota in humans, Spearman’s correlation was conducted for estimated absolute abundances of the genera representing the bacterial strains used in the *in vitro* experiments. The absolute abundance of *Bifidobacterium* was negatively and *Lactobacillus* positively associated with serum ZFPs (*R* = −0.4, *p* < 0.001 and *R* = 0.68, *p* < 0.001, respectively; Figure 2). No association was found between *E. coli* or total bacterial count and serum ZFPs.

**Figure 3:**
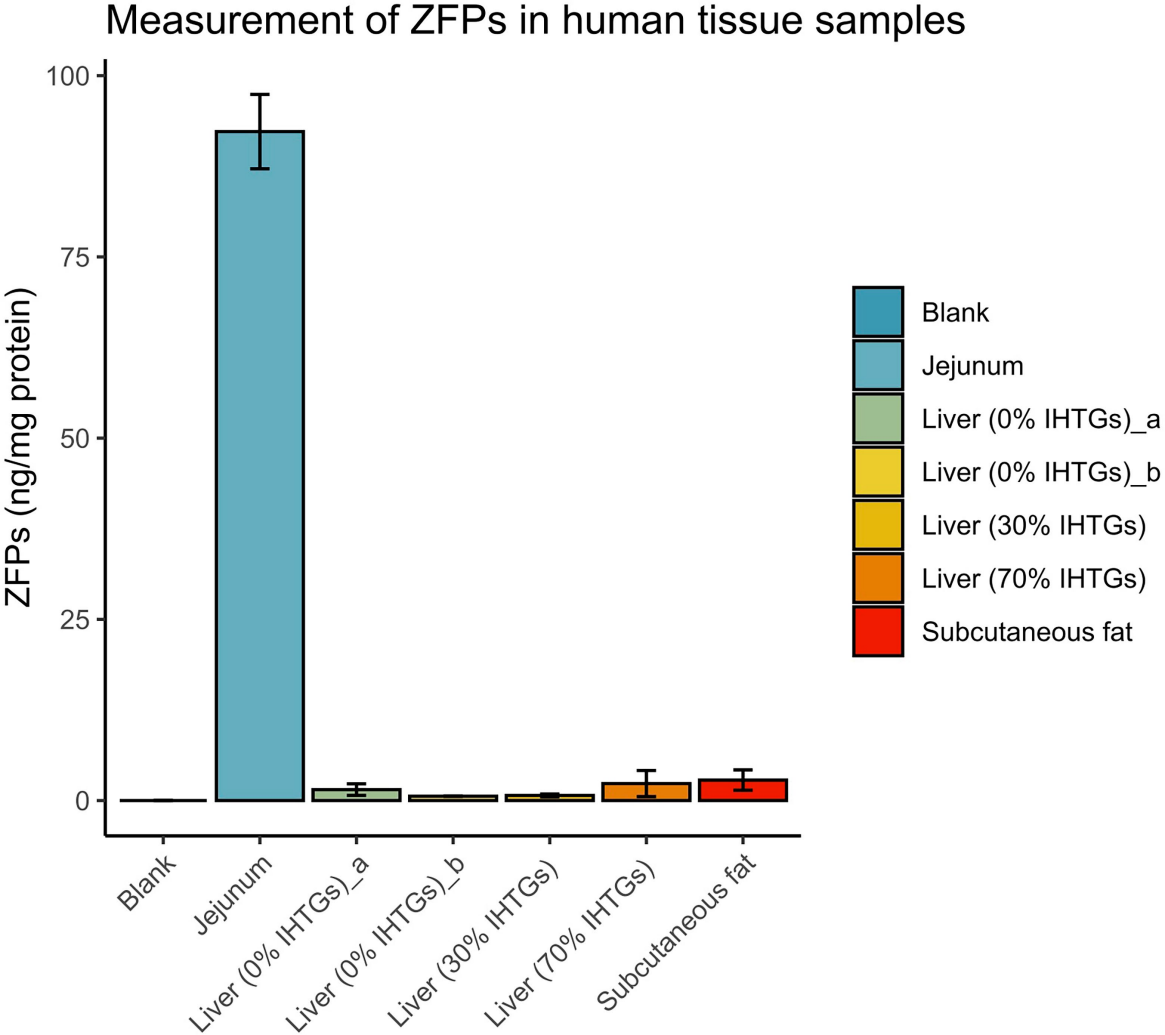
Zonulin family peptide content in extracts of various human tissues. All ZFP concentrations were corrected for the amount of total protein. Error bars represent SDs of three technical replicates in the experiments. Blank, negative control processed using the same protocol with other tissue samples. IHTGs, intrahepatic triglycerides.

To explore the presence and concentration of ZFPs in human tissues where zonulin has been previously reported to be present in non-human mammals, we prepared tissue extracts as described in the section “Materials and Methods” and quantified ZFPs by the IDK® Zonulin (Stool) ELISA. All values were corrected for the total protein content of the extracts. Highest levels of ZFPs were found in the jejunum [92.29 ± 5.13 ng/mg (mean ± SD)], while the liver samples, independent of the IHTGs, or subcutaneous fat tissue had low or undetectable levels of ZFPs (Figure 3).

## Discussion

Touted as the only known endogenous negative modulator of intestinal tight junctions and the only circulating marker of intestinal permeability (Sturgeon and Fasano, 2016), zonulin (pre-haptoglobin 2) has been of great interest since its discovery by Dr. Alessio Fasano and his team 2 decades ago (Wang et al., 2000). Recent studies suggest that commercially available ELISAs measuring zonulin capture unknown proteins, with properdin (complement factor P; Scheffler et al., 2018) and complement C3 (Ajamian et al., 2019) being identified as the potential targets. This discrepancy and/or cross-reactivity are likely due to potential biological or technical variation in the sequence of the epitope chosen to generate the capture antibody in commercially available ELISAs (Scheffler et al., 2018), and that pre-haptoglobin 2 and certain complement-activating proteins are descendants of a common ancestral protein (Kurosky et al., 1980). Subsequently, Dr. Alessio Fasano proposed that the commercially available ELISAs measure a family of structurally and functionally related proteins, ZFPs (Fasano, 2021). With little information about ZFPs, commercial zonulin ELISAs, especially IDK® ELISA kits, continue to be widely utilized in various areas of research (>200 publications to date; Ajamian et al., 2019 and Supplementary Table 1). Here, we show that the human ZFPs measured by the IDK® ELISA kits are likely characteristically different from zonulin in terms of the anatomical sites and regulation of expression and secretion.

Zonulin secretion and the ensuing opening of the paracellular pathway in the small intestine were reported in a pioneering study by El Asmar et al. (2002) where the secretion of zonulin in the rabbit and rat small intestinal explants and cultured human [Caco2 and rat-derived (IEC6) intestinal epithelial cells were shown to be stimulated by both pathogenic and non-pathogenic Gram-negative bacteria, including innocuous *E. coli* K12 DH5α]. Unexpectedly, we observed that only the probiotic strain LGG induced significant ZFP secretion in human HT-29 cells, while the cells exposed to probiotic *B. bifidum*, flagellated *E. coli* RY13, and non-flagellated *E. coli* K12 DH5α did not secrete detectable levels of ZFPs. Moreover, heat treatment of LGG nullified its stimulatory effect on ZFPs. Therefore, it is likely that bacterial metabolites rather than specific structural components contribute to ZFP secretion induced by the live LGG. As a reference, the cell wall components of lactobacilli, such as peptidoglycan and pili, are known to be potent stimulators of innate immunity (Sashihara et al., 2006; von Ossowski et al., 2013). Taken together, zonulin and ZFPs appear to differ in their response to bacterial stimuli. It is worth noting that previous studies have reported the ability of lactobacilli including LGG to alleviate ZFP secretion in Caco2 and HT-29 cells when the cells were pre-treated or co-incubated with gliadin (Orlando et al., 2014; Xu et al., 2016), known to induce zonulin and increase intestinal permeability (Lammers et al., 2008). As these studies did not study the effect of LGG on ZFPs in the absence of gliadin, our results cannot be directly compared.

Given the modulatory role of the gut microbiota in gut barrier function (Régnier et al., 2021) and the presumed utility of zonulin to serve as a marker of gut permeability, numerous studies have explored whether zonulin and ZFPs (measured by various ELISA kits) associate to features of the gut microbiota both in mice and humans. For instance, recent research in a mouse model of rheumatoid arthritis suggests that the gut microbial dysbiosis impairs gut barrier function in a zonulin-dependent manner (Tajik et al., 2020). Zak-Gołąb et al. (2013) found a significant correlation between total bacteria count in feces and circulating ZFPs in 80 adults. Circulating ZFPs have been positively associated with systemic lipopolysaccharide (LPS) and bacterial extracellular vesicle-associated LPS in humans (Al-Obaide et al., 2017; Tulkens et al., 2020; Saitogullari et al., 2021). The relative abundance of *Faecalibacterium*, known to improve gut barrier (Lopez-Siles et al., 2017), has been negatively associated with circulating ZFPs in women (Mokkala et al., 2016; Mörkl et al., 2018). In the present study, we found a positive correlation between circulating ZFPs quantified by the IDK® ELISA kit and the absolute abundance of *Lactobacillus* in the gut microbiota of adults with overweight and obesity. This mirrors our findings in the *in vitro* model, though the genus *Lactobacillus* is a heterogeneous group containing metabolically different species of lactobacilli. On the other hand, circulating levels of ZFPs were negatively associated with the absolute abundance of genus *Bifidobacterium*, congruent with the finding from a previous study (Janczy et al., 2020).

Zonulin and its precursor haptoglobin are known to be produced mainly in the liver and also in the small intestine (Wang et al., 2000), but also found in various other organs (Vanuytsel et al., 2013). The site of expression for ZFPs has not been studied. As an exploratory experiment, we quantified ZFPs in different human tissue biopsies based on sample availability. We found that levels of ZFPs were low or undetectable in the liver and adipose tissues, but abundant in the jejunum. The absence of detectable ZFPs in the liver samples is not a result of individual variation in haptoglobin genotypes (zonulin is produced only in individuals carrying the haptoglobin 2–1 and 2–2 genotypes), as the ZFPs measured by the IDK® ELISA kit are ubiquitous in individuals with different haptoglobin genotypes (Scheffler et al., 2018; Ajamian et al., 2019). Our results on the lack of detectable ZFPs in liver biopsies with variable fat content suggest that the origin of the ZFPs seems rather intestinal than hepatic in humans. Since complement-associated proteins, including properdin (Mangogna et al., 2020), are normally abundant in the liver, our finding casts doubt on properdin being the main potential ZFPs recognized by the IDK® ELISA kit (Scheffler et al., 2018). Future studies may further quantify the concentrations of the abovementioned proteins relevant for ZFPs, such as haptoglobin, complement C3, and properdin, in the human tissues to understand their relationships with the ZFPs.

Owing to the exploratory nature of the present study, our results should be interpreted with caution. The human colon adenocarcinoma cell line HT-29 used in our study possesses functional Toll-like receptor 4 signaling (Lee et al., 2005; Hiippala et al., 2016) that is potentially important for zonulin secretion (Sturgeon and Fasano, 2016) and has been used to study the effects of *Lactobacillus* strains on gliadin-induced zonulin secretion previously (Xu et al., 2016). Other studies investigating the effects of bacterial exposure on zonulin/ZFP secretion have utilized *ex vivo* small intestines or enterocyte-like Caco-2 cells as *in vitro* models (El Asmar et al., 2002; Orlando et al., 2014; Li et al., 2016). The choice of *in vitro* models may contribute to differences in our findings to some degree, although zonulin is secreted by both enterocytes and intestinal epithelial cells (Wang et al., 2000). While the zonulin receptors system is primarily operative in the small intestine (Fasano, 2008), the functional aspects of zonulin or ZFPs (e.g., modulation of intestinal permeability) are outside the scope of the present study. For the *in vivo* correlation analysis, the associations between ZFPs and the absolute abundance estimates of gut bacteria were not adjusted for other clinical characteristics that could potentially confound the relationships. Lastly, due to sample availability, only one jejunal and one subcutaneous adipose tissue samples were used, and all the tissue samples were derived from different individuals. Nevertheless, we included multiple liver samples of varying levels of IHTGs, rendering our finding on the lack of ZFPs in the liver relatively robust.

In conclusion, several characteristics appear to be different between zonulin (pre-haptoglobin 2) and the ZFPs quantified by IDK® Zonulin ELISA kits. The expression of ZFPs is not universally induced by bacterial stimulus in intestinal HT-29 cells but specifically triggered by the exposure to live LGG. The serum levels of ZFPs were positively and negatively associated with the abundance of intestinal *Lactobacillus* and *Bifidobacterium* spp., respectively. Among human tissues, they are copious in the small intestine but undetectable in the liver. Importantly, commercial zonulin assays from different suppliers likely differ in their undisclosed manufacturing processes (e.g., the ELISA kit by CUSABIO; Ajamian et al., 2019), resulting in severe issues in comparability and reproducibility. Therefore, we strongly caution against the quantification of “ZFPs” by commercial assays with any referrals to “zonulin,” as the ZFPs measured by commercial zonulin assays are distinct from zonulin, not thoroughly characterized and defined, and their association to gut permeability remains unknown.

## Data Availability Statement

The original contributions presented in the study are included in the article/**Supplementary Material**; further inquiries can be directed to the corresponding authors.

## Ethics Statement

The studies involving human participants were reviewed and approved by the Medical Ethical Committees of the Hospital District of Helsinki and Uusimaa and HUCH. The patients/participants provided their written informed consent to participate in this study.

## Author Contributions

AS, CJ, and HY-J contributed to conception of the work. CJ, AS, and SK designed the study. CJ and SK performed the experiments and analyzed the data. SQ and HY-J provided the human samples. CJ wrote the manuscript with important inputs from SK and AS. All authors contributed to the article and approved the submitted version.

## Funding

This research was supported by grants from the University of Helsinki (AS), the Otto A. Malm Foundation (CJ), the Academy of Finland (HY-J), the Novo Nordisk Foundation (HY-J), and the Sigrid Jusélius Foundation (HY-J).

## Conflict of Interest

The authors declare that the research was conducted in the absence of any commercial or financial relationships that could be construed as a potential conflict of interest.

## Publisher’s Note

All claims expressed in this article are solely those of the authors and do not necessarily represent those of their affiliated organizations, or those of the publisher, the editors and the reviewers. Any product that may be evaluated in this article, or claim that may be made by its manufacturer, is not guaranteed or endorsed by the publisher.
