# Supplementary material for: In vitro Effects of Bacterial Exposure on Secretion of Zonulin Family Peptides and Their Detection in Human Tissue Samples
Source: Front Microbiol. 2022 Apr 14;13:848128. doi: 10.3389/fmicb.2022.848128 (PMC9048042; doi:10.3389/fmicb.2022.848128)
Supplement: Supplementary file 1 [file Data_Sheet_1.docx]

Supplementary Material

**Supplementary Table 1. Publications measuring serum or fecal ZFPs by commercially available zonulin ELISAs.** Google Scholar and PubMed searched with term “zonulin” for publications between 1.1.2019 and 15.12.2021.The list is not meant to be exhaustive. A list of studies using the Cusabio and Immundiagnostik (IDK^®^) ELISA published before 2019 can be found in the article by Ajamian *et al*. (PMID: 30640940).

| **Assay** | **Author (Year)** | **Title** |
| --- | --- | --- |
| Abcam | Tomsett et al. (2020) | Dietary Fiber Intake Alters Gut Microbiota Composition but Does Not Improve Gut Wall Barrier Function in Women with Future Hypertensive Disorders of Pregnancy |
| Abclonal | Xia et al. (2021) | Elevated Systemic and Intestinal Inflammatory Response Are Associated With Gut Microbiome Disorder After Cardiovascular Surgery |
| ALPCO | Bloomer et al. (2020) | An Assessment of the Glyconutrient AmbrotoseTM on Immunity, Gut Health, and Safety in Men and Women: A Placebo-Controlled, Double-Blind, Randomized Clinical Trial |
| ALPCO | Feng et al. (2021) | Impact of 12-week exercise program on biomarkers of gut barrier integrity in patients with coronary artery disease |
| ALPCO | Vinton et al. (2020) | Biomarkers of Cellular Stress Do Not Associate with sCD14 in Progressive HIV and SIV Infections in Vivo |
| Beijing RGB Technology Development Co. | Meng et al. (2021) | Antibiotics exposure attenuates chronic unpredictable mild stress-induced anxiety-like and depression-like behavior |
| Bio-Synthesis®®® | Maes et al. (2020) | How to Construct a Bottom-Up Nomothetic Network Model and Disclose Novel Nosological Classes by Integrating Risk Resilience and Adverse Outcome Pathways with the Phenome of Schizophrenia |
| Biocompare | Zheng et al. (2021) | Mesenchymal stem cell-derived microvesicles improve intestinal barrier function by restoring mitochondrial dynamic balance in sepsis rats |
| Biomatik | Astudillo-López et al. (2021) | Influence of Diet and Levels of Zonulin, Lipopolysaccharide and C-Reactive Protein on Cardiometabolic Risk Factors in Young Subjects |
| Biomatik | Camara-Lemarroy et al. (2019) | Biomarkers of intestinal barrier function in multiple sclerosis are associated with disease activity |
| Biomatik | Josyabhatla et al. (2021) | Recognizing a MIS-Chievous Cause of Acute Viral Gastroenteritis |
| BioSwamp | Xu et al. (2021) | Altered Fecal Microbiota Composition in Older Adults With Frailty |
| BlueGene | Babu et al. (2021) | Role of zonulin and GLP-1/DPP-IV in alleviation of diabetes mellitus by peptide/polypeptide fraction of Aloe vera in streptozotocin- induced diabetic wistar rats |
| BlueGene | Ehrnthaller et al. (2021) | Hemorrhagic shock induces renal complement activation |
| Cloud-Clone Corp. | Cortez et al. (2020) | Intestinal permeability and small intestine bacterial overgrowth in excess weight adolescents |
| Cusabio | Barbaro et al. (2020) | Serum zonulin and its diagnostic performance in non- coeliac gluten sensitivity |
| Cusabio | Basolo et al. (2020) | Effects of underfeeding and oral vancomycin on gut microbiome and nutrient absorption in humans |
| Cusabio | Chen et al. (2021) | Prevotella copri increases fat accumulation in pigs fed with formula diets |
| Cusabio | de-Faria et al. (2021) | Colonic paracellular permeability and circulating zonulin-related proteins |
| Cusabio | Grimnes et al. (2021) | Impact of a Vancomycin-Induced Shift of the Gut Microbiome in a Gram-Negative Direction on Plasma Factor VIII:C Levels: Results from a Randomized Controlled Trial |
| Cusabio | Lv et al. (2020) | Melatonin Alleviates Neuroinflammation and Metabolic Disorder in DSS-Induced Depression Rats |
| Cusabio | Pearce et al. (2019) | Obesity related metabolic endotoxemia is associated with oxidative stress and impaired sperm DNA integrity |
| Cusabio | Tajik et al. (2020) | Targeting zonulin and intestinal epithelial barrier function to prevent onset of arthritis |
| Cusabio | Wang et al. (2019) | Serum Zonulin in HBV-Associated Chronic Hepatitis, Liver Cirrhosis, and Hepatocellular Carcinoma |
| Cusabio | Wang et al. (2020) | C-type lectin-like receptor 2 and zonulin are associated with mild cognitive impairment and Alzheimer's disease |
| Cusabio | Yin et al. (2021) | Gut-derived lipopolysaccharide remodels tumoral microenvironment and synergizes with PD-L1 checkpoint blockade via TLR4/MyD88/AKT/NF-κB pathway in pancreatic cancer |
| Cusabio | Yuan et al. (2021) | Impaired intestinal barrier function in type 2 diabetic patients measured by serum LPS, Zonulin, and IFABP |
| EASTBIOPHARM | Karagözlü et al. (2021) | The Relationship of Severity of Autism with Gastrointestinal Symptoms and Serum Zonulin Levels in Autistic Children |
| Elabscience | Avşar et al. (2020) | Serum zonulin and claudin-5 levels in children with attention-deficit/hyperactivity disorder |
| Elabscience | Aydın et al. (2020) | Children with Hashimoto’s Thyroiditis Have Increased Intestinal Permeability: Results of a Pilot Study |
| Elabscience | Barillà et al. (2021) | Toll-like receptor 4 activation in platelets from myocardial infarction patients |
| Elabscience | Carnevale et al. (2020) | Low-grade endotoxaemia enhances artery thrombus growth via Toll-like receptor 4: implication for myocardial infarction |
| Elabscience | Carpino et al. (2020) | Increased Liver Localization of Lipopolysaccharides in Human and Experimental NAFLD |
| Elabscience | Cayres et al. (2021) | Detection of Alterations in the Gut Microbiota and Intestinal Permeability in Patients With Hashimoto Thyroiditis |
| Elabscience | Drabińska et al. (2020) | Intestinal Permeability in Children with Celiac Disease after the Administration of Oligofructose-Enriched Inulin into a Gluten-Free Diet—Results of a Randomized, Placebo-Controlled, Pilot Trial |
| Elabscience | Korkmaz et al. (2020) | Could there be a role of serum zonulin increase in the development of hypercalcemia in primary hyperparathyroidism |
| Elabscience | Loffredo et al. (2020) | Is There an Association Between Atherosclerotic Burden, Oxidative Stress, and Gut-Derived Lipopolysaccharides? |
| Elabscience | Loffredo et al. (2020) | Oxidative stress and gut-derived lipopolysaccharides in children affected by paediatric autoimmune neuropsychiatric disorders associated with streptococcal infections |
| Elabscience | Mkumbuzi et al. (2020) | Insulin Resistance is Associated with Gut Permeability Without the Direct Influence of Obesity in Young Adults |
| Elabscience | Pellizoni et al. (2021) | Detection of Dysbiosis and Increased Intestinal Permeability in Brazilian Patients with Relapsing–Remitting Multiple Sclerosis |
| Elabscience | Serrano et al. (2021) | Microbiome as Mediator of Diet on Colorectal Cancer Risk: The Role of Vitamin D, Markers of Inflammation and Adipokines |
| Elabscience | Sirin et al. (2021) | The role of zonulin in the pathogenesis of diabetic retinopathy |
| Elabscience | Trigo et al. (2021) | In vitro digestibility and Caco-2 cell bioavailability of sea lettuce (Ulva fenestrata) proteins extracted using pH-shift processing |
| Elabscience | Usta et al. (2020) | Serum zonulin and claudin‐5 levels in patients with schizophrenia |
| Elabscience | Wang et al. (2020) | Probiotics and fructo-oligosaccharide intervention modulate the microbiota-gut brain axis to improve autism spectrum reducing also the hyper-serotonergic state and the dopamine metabolism disorder |
| Elabscience | Zhang et al. (2021) | Leaky Gut Driven by Dysbiosis Augments Activation and Accumulation of Liver Macrophages via RIP3 Signaling Pathway in Autoimmune Hepatitis |
| Immundiagnostik (IDK®) | Aasbrenn et al. (2020) | Changes in serum zonulin in individuals with morbid obesity after weight-loss interventions: a prospective cohort study |
| Immundiagnostik (IDK®) | Ardehali et al. (2021) | Associations between systemic inflammation and intestinal permeability with Onodera's prognostic nutritional index in critically ill patients |
| Immundiagnostik (IDK®) | Aydın et al. (2020) | Examination of plasma zonulin levels in bipolar I disorder: a case–control study with follow-up |
| Immundiagnostik (IDK®) | Bawah et al. (2020) | Zonulin as marker of pregnancy induced hypertension: a case control study |
| Immundiagnostik (IDK®) | Cano-Ortiz et al. (2020) | Connection between the Gut Microbiome, Systemic Inflammation, Gut Permeability and FOXP3 Expression in Patients with Primary Sjögren’s Syndrome |
| Immundiagnostik (IDK®) | Caviglia et al. (2020) | On-Treatment Decrease of Serum Interleukin-6 as a Predictor of Clinical Response to Biologic Therapy in Patients with Inflammatory Bowel Diseases |
| Immundiagnostik (IDK®) | Caviglia et al. (2021) | Clinical Response and Changes of Cytokines and Zonulin Levels in Patients with Diarrhoea- Predominant Irritable Bowel Syndrome Treated with Bifidobacterium Longum ES1 for 8 or 12 Weeks: A Preliminary Report |
| Immundiagnostik (IDK®) | Cinkajzlová et al. (2019) | Increased intestinal permeability in patients with short bowel syndrome is not affected by parenteral nutrition |
| Immundiagnostik (IDK®) | Coutzac et al. (2020) | Systemic short chain fatty acids limit antitumor effect of CTLA-4 blockade in hosts with cancer |
| Immundiagnostik (IDK®) | Del Bo' et al. (2021) | A polyphenol-rich dietary pattern improves intestinal permeability, evaluated as serum zonulin levels, in older subjects: The MaPLE randomised controlled trial |
| Immundiagnostik (IDK®) | Del Bo' et al. (2021) | Association between Food Intake, Clinical and Metabolic Markers and DNA Damage in Older Subjects |
| Immundiagnostik (IDK®) | Dumitrescu et al. (2021) | Serum and Fecal Markers of Intestinal Inflammation and Intestinal Barrier Permeability Are Elevated in Parkinson’s Disease |
| Immundiagnostik (IDK®) | Farup et al. (2020) | Changes in Faecal Short-Chain Fatty Acids after Weight-Loss Interventions in Subjects with Morbid Obesity |
| Immundiagnostik (IDK®) | Galié et al. (2021) | Effects of the Mediterranean Diet or Nut Consumption on Gut Microbiota Composition and Fecal Metabolites and their Relationship with Cardiometabolic Risk Factors |
| Immundiagnostik (IDK®) | Gargari et al. (2021) | Higher bacterial DNAemia can affect the impact of a polyphenol‐rich dietary pattern on biomarkers of intestinal permeability and cardiovascular risk in older subjects |
| Immundiagnostik (IDK®) | Groele et al. (2021) | Lack of effect of Lactobacillus rhamnosus GG and Bifidobacterium lactis Bb12 on beta-cell function in children with newly diagnosed type 1 diabetes: a randomised controlled trial |
| Immundiagnostik (IDK®) | Haidmayer et al. (2020) | Effects of Probiotic Strains on Disease Activity and Enteric Permeability in Psoriatic Arthritis–A Pilot Open-Label Study |
| Immundiagnostik (IDK®) | Hidalgo-Liberona et al. (2020) | Increased Intestinal Permeability in Older Subjects Impacts the Beneficial Effects of Dietary Polyphenols by Modulating Their Bioavailability |
| Immundiagnostik (IDK®) | Horvath et al. (2019) | Biomarkers for oralization during long-term proton pump inhibitor therapy predict survival in cirrhosis |
| Immundiagnostik (IDK®) | Horvath et al. (2020) | The effects of a multispecies synbiotic on microbiome-related side effects of long-term proton pump inhibitor use: A pilot study |
| Immundiagnostik (IDK®) | Hoshiko et al. (2021) | An Observational Study to Evaluate the Association between Intestinal Permeability, Leaky Gut Related Markers, and Metabolic Health in Healthy Adults |
| Immundiagnostik (IDK®) | Hoshiko et al. (2021) | Exploring the Link between Leaky-Gut-Related Markers and Metabolic Health in a Large Dutch Adult Population |
| Immundiagnostik (IDK®) | Hoshiko et al. (2021) | Identification of leaky gut-related markers as indicators of metabolic health in Dutch adults: The Nutrition Questionnaires plus (NQplus) study |
| Immundiagnostik (IDK®) | Hsu et al. (2021) | Roles and mechanisms of circulating CEACAM1 in the cirrhosis-related intestinal hyperpermeability: in vitro approach |
| Immundiagnostik (IDK®) | Janczy et al. (2020) | Impact of diet and synbiotics on selected gut bacteria and intestinal permeability in individuals with excess body weight – A Prospective, Randomized Study |
| Immundiagnostik (IDK®) | Jung et al. (2021) | Markers of Intestinal Permeability Are Rapidly Improved by Alcohol Withdrawal in Patients with Alcohol-Related Liver Disease |
| Immundiagnostik (IDK®) | Kaczmarczyk et al. (2021) | The gut microbiota is associated with the small intestinal paracellular permeability and the development of the immune system in healthy children during the first two years of life |
| Immundiagnostik (IDK®) | Kaji et al. (2020) | Rifaximin Alleviates Endotoxemia with Decreased Serum Levels of Soluble CD163 and Mannose Receptor and Partial Modification of Gut Microbiota in Cirrhotic Patients |
| Immundiagnostik (IDK®) | Karim et al. (2021) | Intestinal permeability marker zonulin as a predictor of sarcopenia in chronic obstructive pulmonary disease |
| Immundiagnostik (IDK®) | Kartaram et al. (2020) | Kinetics of Physiological Responses as a Measure of Intensity and Hydration Status During Experimental Physical Stress in Human Volunteers |
| Immundiagnostik (IDK®) | Kellerer et al. (2019) | Impact of Laparoscopic Sleeve Gastrectomy on Gut Permeability in Morbidly Obese Subjects |
| Immundiagnostik (IDK®) | Levast et al. (2021) | Impact on the Gut Microbiota of Intensive and Prolonged Antimicrobial Therapy in Patients With Bone and Joint Infection |
| Immundiagnostik (IDK®) | Lingaiah et al. (2021) | Markers of gastrointestinal permeability and dysbiosis in premenopausal women with PCOS: a case–control study |
| Immundiagnostik (IDK®) | Linsalata et al. (2021) | The Relationship between Low Serum Vitamin D Levels and Altered Intestinal Barrier Function in Patients with IBS Diarrhoea Undergoing a Long-Term Low-FODMAP Diet: Novel Observations from a Clinical Trial |
| Immundiagnostik (IDK®) | Łoniewska et al. (2019) | The Influence of Maternal-Foetal Parameters on Concentrations of Zonulin and Calprotectin in the Blood and Stool of Healthy Newborns during the First Seven Days of Life. An Observational Prospective Cohort Study |
| Immundiagnostik (IDK®) | Maget et al. (2021) | Sex differences in zonulin in affective disorders and associations with current mood symptoms |
| Immundiagnostik (IDK®) | Martinez et al. (2020) | Interleukin-10 and Zonulin Are Associated With Postoperative Delayed Gastric Emptying in Critically Ill Surgical Pediatric Patients: A Prospective Pilot Study |
| Immundiagnostik (IDK®) | Meinitzer et al. (2020) | Sex-Specific Associations of Trimethylamine-N-Oxide and Zonulin with Signs of Depression in Carbohydrate Malabsorbers and Nonmalabsorbers |
| Immundiagnostik (IDK®) | Nishioka et al. (2021) | Stratification of Volunteers According to Flavanone Metabolite Excretion and Phase II Metabolism Profile after Single Doses of ‘Pera’ Orange and ‘Moro’ Blood Orange Juices |
| Immundiagnostik (IDK®) | Olsson et al. (2020) | Biomarkers of inflammation and epithelial barrier function in multiple sclerosis |
| Immundiagnostik (IDK®) | Önning et al. (2020) | Intake of Lactiplantibacillus plantarum HEAL9 reduces the inflammatory markers soluble fractalkine and CD163 during acute stress: A randomized, double blind, placebo-controlled study |
| Immundiagnostik (IDK®) | Pietrukaniec et al. (2019) | Zonulin Family Peptide Levels in Ascites and Serum in Patients with Liver Cirrhosis: A Preliminary Study |
| Immundiagnostik (IDK®) | Power et al. (2021) | Serum Zonulin Measured by Commercial Kit Fails to Correlate With Physiologic Measures of Altered Gut Permeability in First Degree Relatives of Crohn’s Disease Patients |
| Immundiagnostik (IDK®) | Prospero et al. (2021) | Psychological and Gastrointestinal Symptoms of Patients with Irritable Bowel Syndrome Undergoing a Low-FODMAP Diet: The Role of the Intestinal Barrier |
| Immundiagnostik (IDK®) | Prospero et al. (2021) | Somatization in patients with predominant diarrhoea irritable bowel syndrome: the role of the intestinal barrier function and integrity |
| Immundiagnostik (IDK®) | Reininghaus et al. (2020) | PROVIT: Supplementary Probiotic Treatment and Vitamin B7 in Depression—A Randomized Controlled Trial |
| Immundiagnostik (IDK®) | Rodriguez et al. (2020) | Metabolite profiling reveals the interaction of chitin-glucan with the gut microbiota |
| Immundiagnostik (IDK®) | Sadowska-Krępa et al. (2021) | Strenuous 12-h run elevates circulating biomarkers of oxidative stress, inflammation and intestinal permeability in middle-aged amateur runners: A preliminary study |
| Immundiagnostik (IDK®) | Sánchez-Alcoholado et al. (2020) | Gut Microbiota-Mediated Inflammation and Gut Permeability in Patients with Obesity and Colorectal Cancer |
| Immundiagnostik (IDK®) | Sánchez-Alcoholado et al. (2021) | Relationships of Gut Microbiota Composition, Short-Chain Fatty Acids and Polyamines with the Pathological Response to Neoadjuvant Radiochemotherapy in Colorectal Cancer Patients |
| Immundiagnostik (IDK®) | Seethaler et al. (2021) | Biomarkers for assessment of intestinal permeability in clinical practice |
| Immundiagnostik (IDK®) | Sjöström et al. (2021) | Increased intestinal permeability in primary Sjögren’s syndrome and multiple sclerosis |
| Immundiagnostik (IDK®) | Søndertoft et al. (2020) | The intestinal microbiome is a co-determinant of the postprandial plasma glucose response |
| Immundiagnostik (IDK®) | Stadlbauer et al. (2020) | Disease severity and proton pump inhibitor use impact strongest on faecal microbiome composition in liver cirrhosis |
| Immundiagnostik (IDK®) | Stadlbauer et al. (2020) | Dysbiosis, gut barrier dysfunction and inflammation in dementia: a pilot study |
| Immundiagnostik (IDK®) | Szymanska et al. (2021) | Fecal Zonulin as a Noninvasive Biomarker of Intestinal Permeability in Pediatric Patients with Inflammatory Bowel Diseases-Correlation with Disease Activity and Fecal Calprotectin |
| Immundiagnostik (IDK®) | Tatucu-Babet et al. (2020) | Serum zonulin measured by enzyme-linked immunosorbent assay may not be a reliable marker of small intestinal permeability in healthy adults |
| Immundiagnostik (IDK®) | Trachtman et al. (2019) | Plasma Zonulin Levels in Childhood Nephrotic Syndrome |
| Immundiagnostik (IDK®) | Utay et al. (2019) | Serum Bovine Immunoglobulins Improve Inflammation and Gut Barrier Function in Persons with HIV and Enteropathy on Suppressive ART |
| Immundiagnostik (IDK®) | Voulgaris et al. (2021) | Serum zonulin levels in patients with liver cirrhosis: Prognostic implications |
| Immundiagnostik (IDK®) | Wegh et al. (2019) | Intestinal Permeability Measured by Urinary Sucrose Excretion Correlates with Serum Zonulin and Faecal Calprotectin Concentrations in UC Patients in Remission |
| Immundiagnostik (IDK®) | Xu et al. (2020) | Inflammatory bowel disease and biomarkers of gut inflammation and permeability in a community with high exposure to perfluoroalkyl substances through drinking water |
| J&L Biological | Wang et al. (2020) | Enhanced therapeutic efficacy of a novel colon-specific nanosystem loading emodin on DSS-induced experimental colitis |
| MyBioSource | Giron et al. (2021) | Plasma Markers of Disrupted Gut Permeability in Severe COVID-19 Patients |
| MyBioSource | Adeniji et al. (2021) | COVID-19 Severity Is Associated with Differential Antibody Fc-Mediated Innate Immune Functions |
| MyBioSource | Barengolts et al. (2019) | Predictors of Obesity among Gut Microbiota Biomarkers in African American Men with and without Diabetes |
| MyBioSource | Brasiel et al. (2021) | Kefir modulates gut microbiota and reduces DMH-associated colorectal cancer via regulation of intestinal inflammation in adulthood offsprings programmed by neonatal overfeeding |
| MyBioSource | Chu et al. (2019) | The Candida albicans exotoxin Candidalysin promotes alcohol-associated liver disease |
| MyBioSource | Edwards et al. (2021) | FPR-1 (Formyl Peptide Receptor-1) Activation Promotes Spontaneous, Premature Hypertension in Dahl Salt-Sensitive Rats |
| MyBioSource | Kolyva et al. (2021) | The effect of feeding patterns on serum zonulin levels in infants at 3–4 months of age |
| MyBioSource | Lang et al. (2020) | Intestinal Fungal Dysbiosis and Systemic Immune Response to Fungi in Patients With Alcoholic Hepatitis |
| MyBioSource | Molina-Vega et al. (2020) | Relationship of Zonulin with Serum PCSK9 Levels after a High Fat Load in a Population of Obese Subjects |
| MyBioSource | Nascimento et al. (2021) | High-Fat Diet Induces Disruption of the Tight Junction-Mediated Paracellular Barrier in the Proximal Small Intestine Before the Onset of Type 2 Diabetes and Endotoxemia |
| MyBioSource | Nelson et al. (2021) | The gut microbiome contributes to blood-brain barrier disruption in spontaneously hypertensive stroke prone rats |
| MyBioSource | Omar et al. (2021) | Effects of High Intakes of Fructose and Galactose, with or without Added Fructooligosaccharides, on Metabolic Factors, Inflammation, and Gut Integrity in a Rat Model |
| MyBioSource | Pražnikar et al. (2021) | Effects of kefir or milk supplementation on zonulin in overweight subjects |
| MyBioSource | Selma-Royo et al. (2021) | Maternal diet during pregnancy and intestinal markers are associated with early gut microbiota |
| MyBioSource | Shin et al. (2020) | Effects of treadmill exercise on the regulation of tight junction proteins in aged mice |
| MyBioSource | Yeung et al. (2021) | Effects of Vitamin D-Deficient Diet on Intestinal Epithelial Integrity and Zonulin Expression in a C57BL/6 Mouse Model |
| N/A (meta-analysis) | Ahmadi et al. (2020) | The Effects of Probiotic/Synbiotic on Serum Level of Zonulin as a Biomarker of Intestinal Permeability: A Systematic Review and Meta-Analysis |
| Promocell | Dirajlal-Fargo et al. (2020) | Micronutrients, metabolic complications and inflammation in Ugandan children with HIV |
| R&D Systems | Alvarez-Mon et al. (2021) | Blunted Expansion of Regulatory T Lymphocytes Is Associated With Increased Bacterial Translocation in Patients With Major Depressive Disorder |
| R&D Systems | Diza et al. (2021) | Systemic Inflammation and the Breakdown of Intestinal Homeostasis Are Key Events in Chronic Spinal Cord Injury Patients |
| R&D Systems | Ho et al. (2021) | Circulating bioactive bacterial DNA is associated with immune activation and complications in common variable immunodeficiency |
| R&D Systems | Li et al. (2021) | IL‑6/STAT3 signaling pathway regulates the proliferation and damage of intestinal epithelial cells in patients with ulcerative colitis via H3K27ac |
| R&D Systems | Mooren et al. (2020) | Effects of Escherichia coli strain Nissle 1917 on exercise-induced disruption of gastrointestinal integrity |
| R&D Systems | Nam et al. (2020) | Regulatory effects of Lactobacillus plantarum HY7714 on skin health by improving intestinal condition |
| R&D Systems | Xu et al. (2019) | Microbial Mechanistic Insights into the Role of Sweet Potato Vine on Improving Health in Chinese Meishan Gilt Model |
| Sun-Red Bio Company | Güvey et al. (2021) | How Do Serum Zonulin Levels Change in Gestational Diabetes Mellitus, Pregnancy Cholestasis, and the Coexistence of Both Diseases? |
| Sunred Biological Technology | Calgin et al. (2019) | Decreased levels of serum zonulin and copeptin in chronic Hepatitis-B patients |
| Thermo Scientific | Duan et al. (2020) | Activated Drp1-mediated mitochondrial ROS influence the gut microbiome and intestinal barrier after hemorrhagic shock |
| Unspecified | Utay et al. (2020) | Evaluation of Six Weekly Oral Fecal Microbiota Transplants in People with HIV |
| Unspecified | Yamaide et al. (2020) | Serum Zonulin levels are higher in pediatric allergic patients than that in healthy children |
| Unspecified | Yüksel et al. (2020) | Measurement of the serum zonulin levels in patients with acne rosacea |
| Unspecified | Zhang et al. (2021) | Antibiotic-Induced Gut Microbiota Dysbiosis Damages the Intestinal Barrier, Increasing Food Allergy in Adult Mice |
| USCN Life Science Co. | Zhang et al. (2021) | Depletion of gut secretory immunoglobulin A coated Lactobacillus reuteri is associated with gestational diabetes mellitus-related intestinal mucosal barrier damage |
| ZellBio | Ghavami et al. (2021) | Effect of synbiotic supplementation on migraine characteristics and inflammatory biomarkers in women with migraine: Results of a randomized controlled trial |

**Supplementary Table 2. Summary of clinical, demographic and gut microbial characteristics of subjects.** Data are in n (%), means ± SEM or median (25th-75th percentile), as appropriate.

| **Variable** | **Value** |
| --- | --- |
| *N* | 38 |
| Age (years) | 48 ± 2 |
| Gender (n, women/men) | 21/17 |
| BMI (kg/m^2^) | 31 ± 1 |
| *Zonulin family peptides (ng/ml) | 32.37 (27.57 - 40.11) |
| **Bifidobacterium* (log copies per g feces) | 9.53 (7.38 - 9.99) |
| **Lactobacillus* (log copies per g feces) | 6.18 (0 - 8.15) |
| **Escherichia coli* (log copies per g feces) | 6.2 (0 - 7.23) |
| *Total bacteria (log copies per g feces) | 11.84 (11.22 - 12.19) |

*Values calculated from 76 samples collected at two time points.


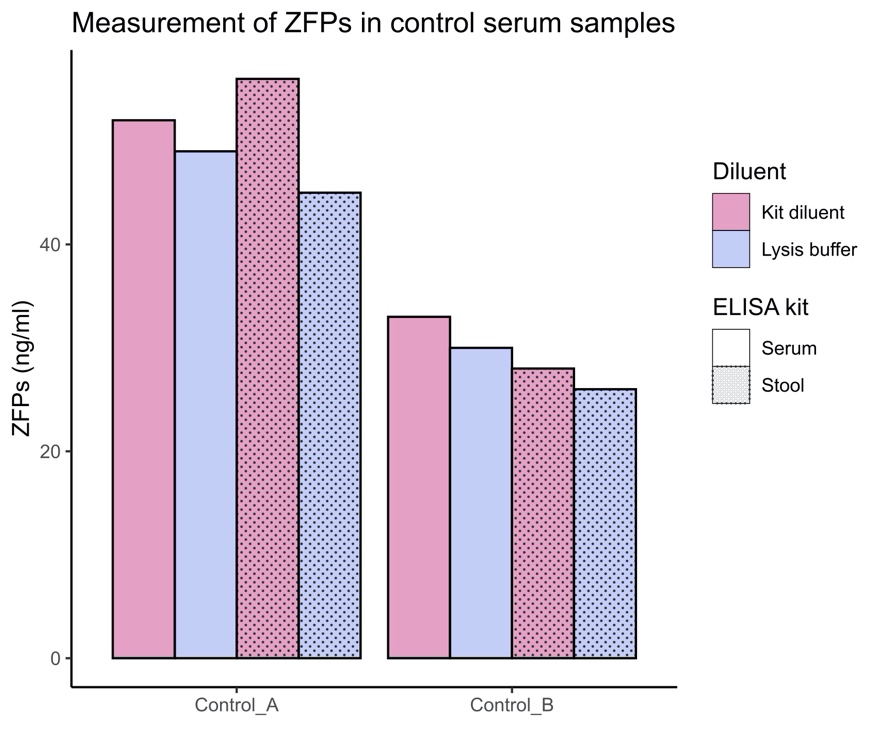


**Supplementary Figure 1.** Effects of technical factors on measurement of serum ZFPs. Two control serum samples (control_A & control_B) were processed according to the same protocol (IDK^®^ Zonulin ELISA), but diluted (1:20) in the assay diluent from the ELISA kit (pink) or the lysis buffer for human tissue samples (purple). To ensure the comparability of the IDK^®^ serum and stool kits, the diluted serum samples were quantified by the serum or stool kit following the same protocol. Neither the diluents nor the kits had discernible effects on serum ZFP measurement.
